# Standardization of Epidemiological Surveillance of Group A Streptococcal Cellulitis^[Author-notes ofac267-FM1]^

**DOI:** 10.1093/ofid/ofac267

**Published:** 2022-09-15

**Authors:** Kate M Miller, Theresa Lamagni, Roderick Hay, Jeffrey W Cannon, Michael Marks, Asha C Bowen, David C Kaslow, Thomas Cherian, Anna C Seale, Janessa Pickering, Jessica N Daw, Hannah C Moore, Chris Van Beneden, Jonathan R Carapetis, Laurens Manning

**Affiliations:** Wesfarmers Centre of Vaccines and Infectious Diseases, Telethon Kids Institute, University of Western Australia, Nedlands, Western Australia, Australia; UK Health Security Agency, London, United Kingdom; St John’s Institute of Dermatology, King’s College London, London, United Kingdom; Wesfarmers Centre of Vaccines and Infectious Diseases, Telethon Kids Institute, University of Western Australia, Nedlands, Western Australia, Australia; Department of Global Health and Population, Harvard T.H. Chan School of Public Health, Boston, Massachusetts, USA; Clinical Research Department, Faculty of Infectious Diseases, London School of Hygiene & Tropical Medicine, London, United Kingdom; Hospital for Tropical Diseases and Division of Infection and Immunity, University College London Hospitals, London, United Kingdom; Division of Infection and Immunity, University College London, London, United Kingdom; Wesfarmers Centre of Vaccines and Infectious Diseases, Telethon Kids Institute, University of Western Australia, Nedlands, Western Australia, Australia; Department of Infectious Diseases, Perth Children’s Hospital, Nedlands, Western Australia, Australia; Faculty of Health and Medicine, University of Western Australia, Nedlands, Western Australia, Australia; PATH, Seattle, Washington, USA; MMGH Consulting, Geneva, Switzerland; UK Health Security Agency, London, United Kingdom; London School of Hygiene & Tropical Medicine, London, United Kingdom; University of Warwick, Coventry, United Kingdom; Wesfarmers Centre of Vaccines and Infectious Diseases, Telethon Kids Institute, University of Western Australia, Nedlands, Western Australia, Australia; Wesfarmers Centre of Vaccines and Infectious Diseases, Telethon Kids Institute, University of Western Australia, Nedlands, Western Australia, Australia; Wesfarmers Centre of Vaccines and Infectious Diseases, Telethon Kids Institute, University of Western Australia, Nedlands, Western Australia, Australia; CDC Foundation, Atlanta, Georgia, USA; Wesfarmers Centre of Vaccines and Infectious Diseases, Telethon Kids Institute, University of Western Australia, Nedlands, Western Australia, Australia; Faculty of Health and Medicine, University of Western Australia, Nedlands, Western Australia, Australia; Wesfarmers Centre of Vaccines and Infectious Diseases, Telethon Kids Institute, University of Western Australia, Nedlands, Western Australia, Australia; Infectious Diseases Department, Fiona Stanley Hospital, Perth, Western Australia, Australia; School of Medicine and Pharmacology, Harry Perkins Research Institute, Fiona Stanley Hospital, University of Western Australia, Perth, Western Australia, Australia

**Keywords:** cellulitis, epidemiology, infectious disease, *Streptococcus pyogenes*, surveillance

## Abstract

Cellulitis is an acute bacterial infection of the dermis and subcutaneous tissue usually found complicating a wound, ulcer, or dermatosis. This article provides guidelines for the surveillance of cellulitis. The primary objectives of cellulitis surveillance are to (1) monitor trends in rates of infection, (2) describe the demographic and clinical characteristics of patients with cellulitis, (3) estimate the frequency of complications, and (4) describe the risk factors associated with primary and recurrent cellulitis. This article includes case definitions for clinical cellulitis and group A streptococcal cellulitis, based on clinical and laboratory evidence, and case classifications for an initial and recurrent case. It is expected that surveillance for cellulitis will be for all-cause cellulitis, rather than specifically for Strep A cellulitis. Considerations of the type of surveillance are also presented, including identification of data sources and surveillance type. Minimal surveillance necessary for cellulitis is facility-based, passive surveillance. Prospective, active, facility-based surveillance is recommended for estimates of pathogen-specific cellulitis burden. Participant eligibility, surveillance population, and additional surveillance considerations such as active follow-up of cases, the use of International Classification of Disease diagnosis codes, and microbiological sampling of cases are discussed. Finally, the core data elements to be collected on case report forms are presented.

## DISEASE CHARACTERISTICS

Cellulitis is an acute bacterial infection of the skin and the subcutaneous tissue commonly affecting lower limbs. It is a diffuse, spreading infection characterized by redness (erythema), warmth (to touch), swelling or edema, and localized pain or tenderness [1]. Fever, chills, and malaise may be present and can be accompanied by lymphangitis and/or bacteremia [1]. Cellulitis usually develops as a result of infection of burns, wounds, surgical incisions, or skin lesions [1, 2]. In adults, cellulitis usually affects the skin on the lower legs and arms but can occur on any part of the body. Among children, cellulitis typically includes infection of the extremities, periorbital infection, and lower limbs [3]. Cellulitis can be caused by multiple bacteria, most commonly *Streptococcus pyogenes* (Strep A), *Staphylococcus aureus*, or other beta-hemolytic streptococci.

The Global Burden of Disease project estimated that almost 43 million cases of cellulitis occurred in 2019 (555 cases per 100 000 population), causing 18 069 deaths [4]. The incidence of cellulitis generally increases with age, with incidence rates highest among older adults [5–9]. Although most cases of cellulitis can be managed by general practitioners, cellulitis can lead to bacteremia and deep tissue infections (eg, septic thrombophlebitis, necrotizing fasciitis, osteomyelitis, abscesses, and infective endocarditis) that require hospitalization [1, 10]. Cellulitis is a common cause of hospital admissions [3, 11, 12].

Clinical diagnosis of cellulitis is based on patient history and physical examination [13]; however, diagnostic accuracy has been shown to vary according to the provider’s clinical specialty and experience [14–16]. Microbiological tests are necessary to confirm Strep A as the cause of cellulitis. However, such testing is not usually indicated as part of routine clinical care, and detection of the etiologic pathogen is challenging; treatment is typically empiric. Diagnostic testing may include bacterial culture (eg, from abscess, wound, or ulcer samples), blood culture, or, less commonly, acute and convalescent antibody detection tests for evidence of recent bacterial infection [17].

With antibiotic treatment, cellulitis usually has a good prognosis [18]. Recurrent cellulitis is a common and challenging problem, and it has been observed in 22% to 49% of adult patients presenting with cellulitis [13]. Risk factors for recurrent cellulitis include lymphedema, dermatophyte infections, obesity, peripheral artery disease, and chronic lower extremity edema (from venous insufficiency, congestive heart failure, hepatic disease, or nephrotic syndrome) [19]. Data on recurrent cellulitis among children are scarce; however, a recent study reported that recurrent cellulitis does occur in children, but less frequently than in older adults [3]. Preventing recurrent episodes has been identified as the key research priority for both clinicians and patients [20].

## OBJECTIVES OF SURVEILLANCE FOR CELLULITIS

An effective surveillance system for cellulitis serves to: (1) monitor trends in age- and sex-specific incidence or prevalence of cellulitis among the population of a defined geographic area; (2) describe the demographic and clinical characteristics of people with confirmed Strep A cellulitis; and (3) provide estimates of disease burden of Strep A cellulitis.

Potential additional objectives that may be incorporated include the following: (1) estimate the frequency of complications of cellulitis (eg, lymphangitis and necrotizing fasciitis); (2) describe the risk factors associated with primary and recurrent cellulitis; and (3) monitor the impact of prevention strategies and interventions on rates of hospitalization for primary and recurrent cellulitis and on the severity of episodes (possibly through proxy measures such as length of stay).

Enhanced (nonroutine) surveillance programs, vaccine trials, or research projects may aim to describe selected genotypic or phenotypic features of Strep A isolates (strains) causing cellulitis (ie, *emm* types, presence of vaccine antigens, and antimicrobial susceptibility) to (1) measure strain-specific disease burden, (2) identify strain-specific outbreaks, (3) predict (evaluate) the effectiveness of prospective (existing) vaccines, (4) monitor temporal trends in Strep A strains causing cellulitis, and (5) track antimicrobial resistance among Strep A isolates over time. These enhanced surveillance objectives are optional and not required in every surveillance system.

## CASE DEFINITIONS AND CASE CLASSIFICATION

Standardized case definitions are important for obtaining robust surveillance data, comparing burden estimates and monitoring the impact of vaccines and other interventions. The definitions and methods presented here may also be used as clinical endpoints for vaccine efficacy trials and for postlicensure effectiveness studies.

Identification of microbial cause in cases of cellulitis is often difficult. Therefore, we propose the following definitions of cellulitis. Classifying cellulitis as an initial or recurrent case is useful in understanding the proportion of the population affected and in tracking the success of primary and secondary prevention programs. See Table 1 for recommended definitions and classifications.

**Table 1.** Case Definitions and Classification of Cellulitis for Surveillance

**Case Definitions**
***Clinical cellulitis***: A case of clinical cellulitis is defined as an infection of the skin manifested by Acute onset (<3 days) and Hot (local warmth), erythematous (red), swollen and tender skin, for which other causes of erythema and tender inflamed skin (eg, deep vein thrombosis, acute lipodermatosclerosis) have been excluded.
***Strep A* (Streptococcus pyogenes) *cellulitis*:** A case of Strep A cellulitis is defined as clinical cellulitis with laboratory-confirmation of Strep A as the etiology by one of the following: Strep A isolated from culture obtained from the affected site or blood culture OR A positive Strep A antibody detection test defined as either: A 2-fold or greater rise in antistreptolysin O (ASO) or anti-deoxyribonuclease B (ADB) titer in specimens collected at least 2 weeks apart (and preferably 4 weeks apart), with the first sample taken within 1 week of symptom onset. OR A single sample taken at least 2 weeks after the onset of cellulitis that is above the upper limit of normal*.
**Case Classifications**
***Initial case*:** A case is considered an initial case if the patient has never been diagnosed with a confirmed case of cellulitis previously. ***Recurrent case*:** It is recommended that an episode is considered a separate but recurrent case of cellulitis if more than 28 days have occurred after the onset of symptoms from a previous episode of cellulitis.

Further information related to the interpretation of serology results is available in Supplementary Appendix 1.

### Notes About Case Definitions

If cellulitis is accompanied by bacteremia or if Strep A is isolated from a deep tissue aspirate or specimen taken from a normally sterile body site, the illness should also be considered an invasive Strep A infection. A case would still be considered Strep A cellulitis if other bacteria are “also” present (eg, *S aureus*).

## SPECIMEN COLLECTION AND LABORATORY TESTS USED FOR DETECTION OF STREP A

Microbiological tests are necessary to confirm Strep A as the etiological agent of cellulitis. However, identification of a causative bacteria through traditional culture methods—whether blood, swabs from skin lesions, needle aspiration, or punch biopsy—is not indicated in the routine clinical care of cellulitis due to the difficulty in obtaining viable cultures, low yield of available tests, and because the results often have no impact on the treatment plan [2, 13]. There are 2 diagnostic testing methods currently available for detection of Strep A in patients with cellulitis: bacterial culture and antibody detection tests. Note that molecular diagnostics (eg, polymerase chain reaction) have yet to prove clinically useful [21, 22].

### Bacterial Culture

Viable culture of Strep A is best taken from the broken skin surface of visible lesions (see Supplementary Appendix 2 for details on specimen collection and laboratory methods). However, in most cases of cellulitis, the skin surface is usually intact. Cultures can be taken by swabbing the surface of the affected site; however, cultures of skin specimens are usually negative. Obtaining cultures subcutaneously, via aspiration or biopsy, are relatively invasive, for typically low yield and are not recommended for the purpose of surveillance.

In clinical practice, blood cultures are only recommended in patients with malignancy, severe systemic features (such as high fever and hypotension), and unusual predisposing factors, such as immersion injury, animal bites, neutropenia, and severe cell-mediated immunodeficiency [23]. Note that Strep A detected in the blood would indicate an invasive Strep A infection.

### Antibody Detection Tests

Antibody detection tests can increase the diagnostic rate but are similarly not conducted as part of routine clinical assessment. For active surveillance within a clinical study, for example, antibody detection tests may be conducted to confirm a case of Strep A cellulitis by demonstrating a positive antibody response to recent Strep A infection. Currently, just 2 titers are used: antistreptolysin O (ASO) and anti-deoxyribonuclease B (ADB). It is recommended that ASO and ADB titers are interpreted by comparing acute and convalescent samples, demonstrating a rise in titer between these 2 time points. Acute serum should be collected as soon as possible after presentation, and convalescent samples should align with the peak antibody titers (ASO and ADB) to optimize sensitivity. Because the timing of the rise for ASO and ADB differ slightly at 3–5 weeks and 6–8 weeks, respectively (although this has been shown to vary between individuals), it is recommended that convalescent samples be conducted between 4 and 6 weeks to best capture the rise across both titers (see Supplementary Appendix 1 for further information). A 4-fold increase in titer from acute to convalescent (taken at least 2 weeks apart and preferably 4–6 weeks apart) is considered the gold standard, however a 2-fold increase is considered acceptable.

## TYPES OF SURVEILLANCE

The selection of surveillance strategies depends on specific epidemiologic and clinical characteristics of the disease outcome of interest, the overall surveillance objectives, surveillance location, services’ accessibility, and the resources available (see Supplementary Appendix 3 for surveillance definitions). A quality management plan should be written before the start of surveillance to establish and ensure the quality of processes, data, and documentation associated with surveillance activities. Furthermore, all surveillance should be conducted in accordance with ethical guidelines (see Supplementary Appendix 4). The minimal and enhanced surveillance strategies for cellulitis are described in Table 2.

**Table 2.** Surveillance Strategies for Cellulitis

***Minimal surveillance***
Minimal surveillance for cellulitis is facility-based, passive surveillance. Passive surveillance is based on identification of diagnosis codes indicating cellulitis as a primary or secondary discharge diagnosis from review of hospital discharge datasets. Because microbiological confirmation of the etiology of cellulitis is not routinely clinically indicated, the objective of passive surveillance is to detect and report on all-cause cases of cellulitis. Cases are identified from ICD codes or location-specific diagnosis codes and are therefore not typically pathogen specific. Passive surveillance identifies people with cellulitis who present on their own accord to healthcare facilities, where the disease is diagnosed by a healthcare provider through routine clinical care and is then recorded. Hospital settings are most commonly used. Primary care facilities with effective electronic medical record systems (EMRs) may also be used if EMRs are used consistently and data are representative of those who access the primary healthcare facility(s). Passive surveillance is best suited to situations where a minimum disease burden estimate is considered adequate for surveillance purposes, and the access and utilization of health services in the catchment population is high. Standard case report forms can be provided to the health facilities for completion and submission to the surveillance programme.
***Enhanced surveillance***
Enhanced surveillance allows for estimates of pathogen-specific cellulitis burden (ie, Strep A cellulitis). Recommended enhanced surveillance for cellulitis comprises prospective, active, facility-based surveillance. Active and timely detection of new cases allows for collection of additional information about healthcare presentations, outcomes, and clinical history. This is important because cases can be misdiagnosed, particularly because there are a number of cellulitis mimics (eg, venous eczema, irritant dermatitis) [24]. Surveillance staff follow a thorough and systematic set of investigative methods so that all potential cases of cellulitis are identified when they are initially diagnosed and consistently over time. Well defined microbiological testing protocol should be established before surveillance and remain constant throughout the surveillance period. Due to the difficulties in obtaining viable culture from patients with cellulitis, antibody detection tests may also be required to determine the etiological agent. Surveillance settings include hospitals, primary healthcare or sole sentinel sites, and microbiological laboratories. Maximizing case ascertainment and relevant data collection requires the establishment of an active data flow pipeline. This may include review of a line listing of potential cases from the data source for investigation, regular contact with select nurses and physicians from settings within the surveillance area to identify any new cellulitis infections among patients, routinely visiting and/or contacting key settings, and reviewing information from infection control logs. Data sources are reviewed, and case counts are reported for all time periods, even if this entails null returns (reporting of zero cases for the time period), which will confirm that case finding methods were followed but no cases were detected. Some programs may have an expert in medical diagnosis of cellulitis (usually physicians) to confirm, qualify, and evaluate the diagnostic information collected to ensure the accuracy of the disease code and exclude cellulitis mimics. Training may need to be provided to clinical staff to recognize cellulitis and record it accurately. A key component of enhanced surveillance is regular feedback of information to healthcare workers and others involved in the surveillance process. This critical communication should engage community healthcare workers in the process so that it informs their clinical practice.

### CASE ASCERTAINMENT AND SURVEILLANCE SETTINGS

It is expected that surveillance of cellulitis will be for all-cause cellulitis, rather than specifically for Strep A cellulitis. As a subset, confirmed Strep A cases can be identified. Recognizing the difficulties with obtaining a confirmatory diagnosis, the estimated fraction of cellulitis cases due to Strep A will often need to be inferred using several lines of evidence. Case ascertainment may be active or passive (see Supplementary Appendix 5).

Common data sources used for cellulitis surveillance include hospital records (admission logs and discharge diagnosis), primary care records (doctors’ offices, outpatient and emergency departments), or health insurance databases. Considerations for using administrative health databases to identify cases are provided in Supplementary Appendix 6. More severe cases of cellulitis will present to hospital, in settings where there is access to this care. Where access to care is more limited there will be an underestimation of disease incidence. Surveillance staff may choose to expand surveillance to include community-level care to capture milder cellulitis cases. These will be important in estimating the total burden of cellulitis given that only a proportion will require hospital care. Active community surveillance provides a more comprehensive disease estimate; however, the increases in the number of surveillance sites may substantially increase cost, and the complexity, depending on availability of primary care networks to embed such surveillance. As confirmatory microbiological testing on cellulitis patients may not be routinely performed and/or yield positive results, it is recommended that laboratory-based surveillance be used as an adjunct to other reporting sources rather than a primary source.

For each data source, surveillance staff should (1) know the purpose of the data source (ie, routinely collected as part of patient care, mandatory collection of data under legal mandates, collected for research purposes, other); (2) identify any legal mandates governing the operations of the data source that may impact the accessibility or quality of the data from that source; (3) describe the representative population for the data; and (4) know the limitations of the data (eg, *International Classification of Diseases* [ICD] diagnosis codes for cellulitis are not pathogen-specific).

### SURVEILLANCE POPULATION

A surveillance protocol should clearly describe enrollment eligibility criteria. Persons with underlying immunocompromise, chronic diseases or pregnant or lactating persons should not be excluded from surveillance. The denominator must be well characterized to derive meaningful estimates of disease burden. The surveillance will usually occur in a defined geographic or health facility catchment area, and therefore the denominator must be defined as the total number of eligible at-risk people from which cases are identified.

Facility-based active surveillance can involve a defined population, for example, when it is the only facility in the region, or a select cohort from the catchment population of the health facility. Ideally, in instances when surveillance is based in a sentinel hospital, or where select health facilities serve a portion of a population residing in the geographical catchment area and is thus not population-based, healthcare utilization surveys are used to estimate the denominator that corresponds to the cases of interest. This improves the accuracy of disease burden estimates and enables rate calculations. The denominator is the number of patients within the geographical catchment area who would be expected to attend that facility if signs and symptoms of cellulitis develop. Without an accurate accounting of all people in the sample population that gave rise to the numerator, incidence may be under- or overestimated [23, 25]. If cases do not reside in the defined catchment area, they should be excluded. Ideally, the denominator population should be defined before surveillance begins.

## SPECIAL CONSIDERATIONS FOR CELLULITIS SURVEILLANCE

### *International Classification of Diseases* Diagnosis Codes

The ICD diagnosis codes available in the country of surveillance can be used to identify cellulitis cases. If using the ICD-10 codes, the codes L03.01–L03.91 should be collected to capture the different presentations of cellulitis. Because erysipelas is sometimes used synonymously with cellulitis, the ICD code A46 (erysipelas) may be used to identify probable cases for further investigation (see Supplementary Appendix 7 for a full list of cellulitis-specific ICD codes). Care should be taken to note any subtle differences between international versions of ICD (eg, ICD-10-AM in Australia) because diagnosis codes may be different. Note that none of the cellulitis-specific ICD codes have pathogen-specific subcodes. Therefore, the respective pathogen-specific burden for cellulitis can only be inferred using knowledge about attributable proportions for causative pathogens.

### Cellulitis Mimics

Distinguishing cellulitis from erysipelas and other cellulitis mimics such as infectious and noninfectious conditions such as deep vein thrombosis, acute gout, septic arthritis, dermatitis, and necrotizing soft tissue infection may be difficult [26]. Similarities in the clinical presentation of cellulitis and erysipelas mean that the terms are often used interchangeably, despite different diagnostic codes. Cellulitis typically has poorly defined borders and the erythema is typically pinkish-hued. In contrast, erysipelas, which involves the superficial epidermis, is notable for well demarcated borders of infection and a brilliant red skin color.

### Surveillance Period

Given the lower incidence of cellulitis relative to Strep A pharyngitis and impetigo, longer periods of surveillance are generally required to obtain accurate estimates of incidence and of strain distribution. Several years of surveillance is generally required to monitor recurrent cellulitis, to elucidate year-to-year variations in incidence and *emm* type distribution, and to monitor the impact of public health interventions such as the introduction of a vaccine program [27].

### Season

Seasonality has been reported in some studies, with seasonal peaks in summer months and troughs in cooler months of temperate climates, but this is not the case in all countries. Investigators should conduct surveillance throughout the year to include all seasons and as where applicable. Continuous surveillance over 12 months is optimal; multiple years of surveillance is necessary to describe seasonality.

### Active Follow-up of Cases

The extent of follow-up of patient illness outcome will be determined by the specific protocol. If serological methods are being used, active follow-up will be required up to 4–6 weeks after illness onset for convalescent testing. Studies interested in recurrent cellulitis may actively follow-up cases for several years postdischarge if consent has been obtained from the patient. Recurrence rates are dependent on patient risk factors and the number of previous episodes. However, most recurrent episodes occur within the first 5 years from the last episode [19].

### Microbiological Sampling of Cases

As microbiological testing for cellulitis is uncommon, the potential for bias in interpreting laboratory results should be considered as these may not be representative of the population in terms of etiology or antibiotic resistance profiles. If additional surveillance strategies are embedded to obtain laboratory specimens (including microbiological and serology) for identification of the causative bacteria, physicians should be encouraged to collect specimens for bacterial cultures prior or close to initiation of antibiotic treatment where possible.

### Measurement of Disease Burden

The burden of cellulitis can be described in terms of incidence or prevalence. To enable comparison between different population sizes (eg, between different countries or between different time periods in the same country), the incidence rate of cellulitis should be calculated and reported. An incidence rate is the number of new episodes occurring per person per period of time at risk (person-time). To derive the incidence rate, the number of episodes is divided by the total number of person-time units (eg, person-years) in which all the individuals in the group were under surveillance. The person-time is the total time a person had been observed as being disease-free. Incidence rate calculations should include all potential data sources that service the defined population, ensuring that infections are not counted more than once.

Note that data from those with cellulitis at the start of the study would be considered prevalent cases and can only be included as an incidence case once they become disease-free, and they will be considered to have recurrent cellulitis at the time of their next episode. Given the frequency of cellulitis, it is recommended that incidence be expressed as episodes per 1000 population per year. It is useful to report incidence rates for initial and recurrent episodes as well as clinical and Strep A confirmed cellulitis separately where possible.

## DATA COLLECTION AND CASE REPORT FORMS

Case report forms should be based on collecting only the information required to achieve the surveillance objectives. Supplementary Appendix 8 provides a list of recommended and optional variables for inclusion in case report forms.

General surveillance variables include unique identifier, date and time of first enrollment or specimen collection, and site where participant is seen, such as setting, location, postcode, state/province/region, and country. Each encounter should also record a surveillance visit number/episode number if repeated episodes from the same person are included.

Key demographic variables include date of birth or age (in days or months if <12 months and otherwise in years), sex, ethnicity/race, residential postcode, state/province/region, and country.

Clinical and epidemiologic variables include information on anatomical site, clinical risk factors, severity of illness and disease outcome, presumed portal of Strep A entry (ie, skin trauma or an underlying lesion/presence of a wound, ulcer, or dermatosis), number and timing of previous episodes, epidemiologic risk factors, comorbidities, microbiological results and treatment, and length of stay, if hospitalized. Consideration should be given to capture information that will facilitate assessment of differential risk according to predisposing factors (eg, obesity, chronic skin lesions) or wider determinants of health.

Depending on the purposes of surveillance it may be appropriate to document site of infection. This may include site categories such as lower limb, upper limb, facial, and dental or odontogenic.

If the severity of cellulitis is measured, grading should be conducted using reproducible methods where possible. There are several stratification algorithms used in clinical practice, most of which grade the severity of cellulitis based on the presence or absence of defined systemic features and/or significant comorbidities. Examples include the Eron classification of cellulitis [28] and Dundee criteria [29].

## Supplementary Data

Supplementary materials are available at *Open Forum Infectious Diseases* online. Consisting of data provided by the authors to benefit the reader, the posted materials are not copyedited and are the sole responsibility of the authors, so questions or comments should be addressed to the corresponding author.

## Supplementary Material

ofac267_Supplementary_Data
