# Supplementary material for: Standardization of Epidemiological Surveillance of Group A Streptococcal Cellulitis
Source: Open Forum Infect Dis. 2022 Sep 15;9(Suppl 1):S25–30. doi: 10.1093/ofid/ofac267 (PMC9474943; doi:10.1093/ofid/ofac267)
Supplement: ofac267_Supplementary_Data [file ofac267_supplementary_data.docx]

**Standardization of Epidemiological Surveillance of Group A Streptococcal Cellulitis**

Supplementary Appendices

Table of Contents

[Appendix 1: Antibody Detection Test 2](#_Toc112315267)

[Appendix 2: Microbiological Culture of Specimens to Detect Strep A Among Persons With Cellulitis 3](#_Toc112315268)

[Appendix 3: Definitions of Key Surveillance Terms 5](#_Toc112315269)

[Appendix 4: Good Practice and Ethical Considerations 6](#_Toc112315270)

[Appendix 5: Comparisons of Advantages and Disadvantages of Active and Passive Surveillance 7](#_Toc112315271)

[Appendix 6: Administrative Health Databases 8](#_Toc112315272)

[Appendix 7: ICD Diagnosis Codes for Cellulitis 9](#_Toc112315273)

[Appendix 8. Suggested Variables to be Included in Cellulitis Surveillance Datasets 11](#_Toc112315274)

[References 13](#_Toc112315275)

## Appendix 1: Antibody Detection Test

Interpretation of Strep A serology results can be difficult in communities with high incidence or prevalence of skin or upper respiratory Strep A infections^1^. In these settings, a negative antibody test helps exclude a recent infection, but a positive test does not necessarily indicate an infection in the past few weeks. Wherever possible, titers should be interpreted by comparing acute and convalescent samples and demonstrating a titer rise between these two time-points.

An upper limit of normal (ULN) cut-off (80^th^ percentile) can be used in place of the gold standard four-fold rise in titer when paired sera are not available. Ideally, age stratified ULN values for serum ASO and ADB titers will be available for a subset of healthy individuals, without recent Strep A infection, and drawn from the surveillance population of interest. Local data, where available, should be used to determine threshold titers as values can differ between and within countries based on variables such as ethnicity, geography, and socioeconomic status. However, developing local population ULN values is not always possible due to logistics, cost, or surveillance occurring in regions where streptococcal infections are endemic, and it is difficult to identify local children without a recent infection.

Recommended upper limits of normal for anti-streptolysin O and anti- DNase B titers, in the absence of appropriate local population data.

| **Age group (years)** | **Upper limit of normal (international units/mL)** | |
| --- | --- | --- |
|  | **ASO titer** | **Anti-DNase B titer** |
| 2-4 | 160 | 240 |
| 5-9 | 240 | 320-640 |
| 10-12 | 320 | 480-640 |
| >12 | 400 | 200 |

From: Kaplan EL et al, Pediatrics 1998; 101: 86-8; Gray GC et al. J Clin Epidemiol 1993; 46: 1181-5; and Karmarkar MG et al, Indian J Med Res. 2004;119 Suppl:26-8.

## Appendix 2: Microbiological Culture of Specimens to Detect Strep A Among Persons With Cellulitis

Microbiological culture of specimens is done in a laboratory setting to inform diagnosis, and for antimicrobial susceptibility testing. Typically, clinical lesional skin swabs are inoculated onto blood agar plates, however selective plates can be used.^2^ Inoculated agar plates are initially incubated at 37°C for 18–24 hours, but incubation up to 48 hours may be necessary. The addition of 5–10% CO_2_ for incubation may enhance growth but is not essential. Following incubation, plates are inspected for β-hemolytic colonies to undergo subculture purification and confirmation with further biochemical tests including latex agglutination testing (for Lancefield groups A, C, G), bacitracin sensitivity and PYR testing. No biochemical test is 100% specific for *Streptococcus pyogenes*^3^ and so are frequently used in combination*.* Purified colonies can be stored enabling further testing, with long term storage between -70 and -80°C in a suitable cryoprotectant medium (e.g., in Todd Hewitt Glycerol broth or STGGB). Molecular profiling of *emm* types (via Centers for Disease Control and Prevention methodology or WGS-derived methods^4^), or whole genome sequencing to differentiate Strep A strains, can support surveillance by indicating the diversity of strains in a population over time, and to map transmission in communities.

**Specimen collection**

*Equipment and supplies:*

- Gloves (need not be sterile)
- Sterile swabs (cotton wool or synthetic fiber) and culture medium (e.g., eSwab, STGGB, Amies)
- Sterile normal saline
- Biohazard plastic bags, or clean plastic bags than can be labelled
- Transport container
- Cooling bricks (if refrigerated storage is recommend for choice of culture medium)

*Methods of sample collection*

1. Verify the identity of the person and label a sterile culture swab tube with the information requested by the protocol [typically two case identifiers, such as initials and surveillance number, the date, and an identifier for the person plating the organism] and complete a sample form with participant details.
2. Perform hand hygiene, and if appropriate put on gloves.
3. If a person has multiple lesions, it is recommended that the swab is taken from the main site of infection.
4. Use a sterile swab (packages with intact wrapping), taking care to keep the swab tip sterile after the packaging has been opened.
5. The sample collection method depends on the nature of cellulitis:
   1. For *purulent lesions*, pass the swab over the wound, collecting available pus.
   2. *For lesions without evidence of pus*, the swab should be moistened with sterile water or saline and rolled gently backwards and forwards several times over the site of infection.
   3. *If blisters (bullae) are present,* culture of the blister aspirate fluid may be used*.*
6. Avoid touching normal skin with the swab.

*Storage and handling*

- Make sure the top is screwed on or pushed on firmly in place
- All specimens should be stored in sealed biohazard plastic bags or inside a biohazard labelled sealed container
  - Store at the temperature required by culture medium. For example, room temperature storage is suitable for eSwabs (Copan, Italy), whereas refrigerated (in fridge) conditions are recommended for specimens stored in STGGB.
- Sample collection documentation must be kept with specimens, but not in the same compartment in case of leakage.

*Documentation*

- Label all specimens
  - Follow instructions on sticky label on tube/swab container
  - Minimum information needed
    - Unique participant ID number
    - Date specimen collected
    - Exactly what specimen is (e.g., blood, wound swab)
- A specimen transport log form should be used, consisting of:
  - Place, date, and time of collection shipment
  - Contents of shipment including participant ID numbers, specimen types and order of storage.

*Specimen Transfer*

- Place absorbent material in sealed biohazard bags with specimens in case of sample leakage
- Put into recommended portable transport container. For samples collected into storage medium with refrigeration recommended (i.e., STGGB), store sealed bags in between ice cooler bricks.
- Seal lid of portable container as instructed or with waterproof tape
- Label all containers clearly with:
  - Place, date, time of packing, and destination
  - Biohazard sticker. If no sticker, write it in big letters using black marker
- Make sure the courier knows what contents are, so they will not be left in a hot place and will be promptly delivered to the laboratory.
- Specimens should reach the laboratory as soon as possible (≤10 days).

## Appendix 3: Definitions of Key Surveillance Terms

| **Syndromic surveillance** | Syndromic surveillance refers to the use of a clinical syndrome (a constellation of symptoms and signs) as the case definition for detection of suscept cases. Syndromic surveillance can be used for initial case detection, but laboratory confirmation should occur to increase the accuracy of the system.^8^ |
| --- | --- |
| **Active surveillance** | Active case detection means that designated public health surveillance staff are directly involved in detecting cases.^8^ |
| **Passive surveillance** | Passive case detection means that health facility staff detect and report cases to the public health system.^8^ |
| **Facility-based surveillance** | Facility-based surveillance is based on ascertainment of cases in persons who seek care at health facilities, including outpatient clinics, doctors’ offices, hospitals and emergency departments.^8^ |
| **Sentinel-site surveillance** | Sentinel-site surveillance refers to a system that captures cases at one or more specialized sites, such as hospitals, clinics, schools or pharmacies.^8^ |
| **Community-based surveillance** | Community-based surveillance is the systematic detection and reporting of events of public health significance within a community-by-community members. Community-based surveillance enables earlier detection of the disease of interest and captures illnesses in persons who do not seek care in a hospital.^9^ |
| **Population-based surveillance** | Population-based surveillance attempts to capture all cases in a well-defined catchment population (for example, the entire population of a country). |
| **Healthcare utilization surveys** | Healthcare utilization surveys seek to characterize the health care-seeking behavior of ill persons by describing where ill persons sought health care for their illnesses, and soliciting reasons for not seeking health care.^10^ |
| **Unique identifier** | Unique identifiers are unique numbers or numbers and letter combinations that are allocated to a specific individual person. |

## Appendix 4: Good Practice and Ethical Considerations

**Monitoring/Audit**

A systematic and independent audit of surveillance systems should be undertaken to ensure that surveillance and surveillance-related activities were conducted following the relevant surveillance protocol, SOPs, ethical guidelines, and regulatory requirement(s) established by local public health. Existing surveillance review tools can be modified to guide the investigation (e.g., WHO’s ‘[Tools for a surveillance review: Vaccine Preventable Diseases Surveillance Standards](https://www.who.int/publications/m/item/vaccine-preventable-diseases-surveillance-standards-annex1)’). Surveillance as part of a clinical study should adhere to the International Council for Harmonisation (ICH) Guidelines for Good Clinical Practice ^11^.

**Quality control and quality assurance**

A quality management plan should be written before the start of surveillance to establish and ensure the quality of processes, data, and documentation associated with surveillance activities. It encompasses both quality control (QC) and quality assurance (QA) activities.

Surveillance systems should develop SOPs to ensure confidentiality for all cases, ensure that clinical specimens and bacterial isolates obtained are not compromised by human and processing errors, validate data integrity, and maintain multiple layers of security. A SOP will ideally detail:

- Data storage. Including participants’ unique surveillance ID numbers in each respective dataset enables linkage to other datasets, such as hospital admissions, facilitating the capture of complications and ensuring that all personal identifying information is removed from research/surveillance datasets
- Data evaluation for protocol compliance and source document accuracy
- Document review (e.g., specimen tracking logs, questionnaires), who is responsible, and frequency
- Who the responsible person is for addressing QA issues (correcting procedures that do not comply with the surveillance protocol) and QC issues (correcting errors in data entry)
- Staff training activities and processes for documenting surveillance staff training
- Maintenance and strict adherence to surveillance delegation log (list of staff involved in the surveillance and their duties/roles)
- Clinical and laboratory SOPs and accreditation
- Regular audits of surveillance data to ensure accuracy and completion
- System for periodic and refresher training for surveillance team

**Ethics of surveillance**

The global network of WHO Collaborating Centres for Bioethics in collaboration with the U.S. Centers for Disease Control and Prevention developed ethical guidelines for public health surveillance, including common good, respect for persons, and good governance. The guidelines cover the (i) broad responsibility for undertaking surveillance and subjecting it to ethical scrutiny; (ii) obligation for ensuring appropriate protection and rights; (iii) considerations in making decisions about how to communicate and share surveillance data. The guidelines are available at <https://apps.who.int>. Countries should implement these guidelines and monitor them regularly. As appropriate, surveillance protocols should adhere to existing country-specific ethical guidelines.

## Appendix 5: Comparisons of Advantages and Disadvantages of Active and Passive Surveillance

| **Advantages** | **Disadvantages** |
| --- | --- |
| ***Active surveillance*** |  |
| - Sensitive system that facilitates early detection of new cases - Higher case ascertainment rate - More accurate identification of cases - Ability to verify information in the case of missing data or suspected data entry errors - Data collected can be comprehensive and specific to the surveillance objectives - Can evaluate the quality and effectiveness of case-finding process, thus minimizing selection bias - Allows real-time analysis and ability to respond/modify approach to surveillance and care - Can promote disease awareness and good health practices | - Can be costly and resource-intensive - Requires dedicated surveillance staff and/or extensive training and upskilling - Can be demanding on surveillance sites |
| ***Passive surveillance*** |  |
| - Can be conducted retrospectively - Requires fewer resources than active surveillance - Can support real-time reporting | - Responsibility for reporting new cases lies with healthcare workers/laboratory staff; thus, it can be difficult to ensure compliance by healthcare providers and other reporters - Difficulties caused by lack of standardization in terms of case definitions and coding - Tends to under-report disease - Commonly associated with incompleteness of data recording or of microbiological studies - Often difficult to confirm data recording or entry errors retrospectively |

## Appendix 6: Administrative Health Databases

Administrative data from electronic medical records (EMR) from primary healthcare, emergency departments, and hospitalization episodes that cover whole communities can offer a timely and cost-effective surveillance option.

An important consideration when using EMRs to calculate disease estimates in a population is that the data are collected and coded as part of service delivery or reimbursement rather than for surveillance purposes. Routinely collected clinic data may be insufficient for evaluating potential cases against the full criteria required to meet surveillance case definitions, especially when microbiological testing is not routinely conducted or recorded. For EMRs that include or rely on free text, new methods in machine learning or deep learning could improve case identification.^5,6^ Data may be insufficient for addressing other surveillance objectives, such as strain typing and antimicrobial susceptibility testing. Despite the limitations of EMRs, when used consistently across health services data, EMRs can provide important surveillance data on disease burden, including populations at high risk, trends over time, geographic and seasonal patterns, and service utilization due to cellulitis. EMRs can form the basis of enhanced surveillance by using an additional data collection form to augment routinely collected data. The use of common case definitions, coding practices, and protocols for bacterial confirmation can improve the value of EMR data.

EMR databases can vary in the number of diagnoses that can be recorded. Many hospital admission databases contain a principal or primary diagnosis as the main reason for hospital admission and several additional diagnosis codes. Consequently, analysts should check all diagnosis fields, with secondary diagnosis or comorbid cases of cellulitis (i.e., not principal diagnosis) considered a case and included in the numerator. Efforts should be made to validate diagnosis ^7^ and assess data quality of administrative data. Record linkage of administrative datasets can improve case ascertainment rates, identify missed cases, monitor co-morbidities and be useful for monitoring recurrence, complications, and case fatality.

## Appendix 7: ICD Diagnosis Codes for Cellulitis

Note that none of the cellulitis-specific ICD codes have pathogen specific sub-codes to indicate Strep A as the etiology. ICD-10 diagnosis codes listed here are current as of February 2021 and should be used as a guide only.

**L03** – Cellulitis and acute lymphangitis

**L03.0** – Cellulitis and acute lymphangitis of finger and toe

**L03.01** – Cellulitis of finger

- **L03.011** – Cellulitis of right finger
- **L03.012** – Cellulitis of left finger
- **L03.019** – Cellulitis of unspecified finger

**L03.03** – Cellulitis of toe

- **L03.031 – Cellulitis of right toe**
- **L03.032 – Cellulitis of left toe**
- **L03.039 – Cellulitis of unspecified toe**

**L03.1** – Cellulitis and acute lymphangitis of other parts of limb

**L03.11** – Cellulitis of other parts of limb

- **L03.111 – Cellulitis of right axilla**
- **L03.112 – Cellulitis of left axilla**
- **L03.113 – Cellulitis of right upper limb**
- **L03.114 – Cellulitis of left upper limb**
- **L03.115 – Cellulitis of right lower limb**
- **L03.116 – Cellulitis of left lower limb**
- **L03.119 – Cellulitis of unspecified part of limb**

**L03.2** – Cellulitis and acute lymphangitis of face and neck

**L03.21** – Cellulitis and acute lymphangitis of face

- **L03.211 – Cellulitis of face**
- **L03.213 – Periorbital cellulitis**

**L03.22** – Cellulitis and acute lymphangitis of neck

- **L03.221 – Cellulitis of neck**

**L03.3** – Cellulitis and acute lymphangitis of trunk

**L03.31** – Cellulitis of trunk

- **L03.311 – Cellulitis of abdominal wall**
- **L03.312 – Cellulitis of back [any part except buttock]**
- **L03.313 – Cellulitis of chest wall**
- **L03.314 – Cellulitis of groin**
- **L03.315 – Cellulitis of perineum**
- **L03.316 – Cellulitis of umbilicus**
- **L03.317 – Cellulitis of buttock**
- **L03.319 – Cellulitis of trunk, unspecified**

**L03.8** – Cellulitis and acute lymphangitis of other sites

**L03.81** – Cellulitis of other sites

- **L03.811 – Cellulitis of head [any part, except face]**
- **L03.818 – Cellulitis of other sites**

**L03.9** – Cellulitis and acute lymphangitis, unspecified

- **L03.90 – Cellulitis, unspecified**

## Appendix 8. Variables for Inclusion in Cellulitis Surveillance Datasets

| **Category of variables** | **Required variables** | **Optional variables** |
| --- | --- | --- |
| **General** | - Unique ID number - Date of enrolment to surveillance/study | - Illness onset date |
| **Demographics** | - Age (in months if <1 year; otherwise in years) - Sex | - Date of birth - Race/ethnicity - Residential address |
| **Diagnosis** | - ICD 10 code or physician diagnosis | - Microbiologically confirmed Strep A (if routinely performed as part of surveillance)* - Anatomical site involved |
| **Clinical risk factors for cellulitis** |  | - AIDS or CD4 count <200 - HIV infection - Underlying chronic illness - Underlying immunosuppression condition - Diabetes - Skin barrier disruption due to trauma (such as abrasion, penetrating wound, pressure ulcer, venous leg ulcer, insect bite, injection drug use) - Chronic skin lesion (e.g., leg ulcers) - Skin inflammation (such as eczema, radiation therapy, psoriasis) - Edema due to impaired lymphatic drainage - Edema due to venous insufficiency - Pre-existing skin infection (such as tinea pedis, impetigo, varicella) - Prior saphenous vein harvesting for coronary artery bypass graft surgery Inflammation (e.g., eczema) - Obesity |
| **Epidemiologic risk factors** |  | - Intravenous drug use - Older age - Homelessness - LTCF residence - Recurrent impetigo |
| **Severity of disease and outcome** |  | - Hospitalized - Death (within 30 days) - ICU (yes/no; number of days) - Debridement - Discharged to (home; rehab; other__) - Other_____ (*describe*) |
| **Treatment** |  | *During hospitalization*:   - Date of admission - Date of discharge - Admitted to ICU - Length of stay - Antibiotics (route of administration; name of antibiotic; duration; dose) - β-lactam alone, β-lactam + clindamycin, β-lactam + other, non- β-lactam |
| **Microbiology** | - Participant unique ID number* - Specimen unique IDǂ - Specimen collection date - Date and hour plate is inoculated - Date and hour plate is placed in incubator - Date(s) and hour(s) plate is read - Name of reporting laboratory - Laboratory ID (if present)* - Episode number (if repeated episodes from the same person are included) - Site from which organism isolated: type (or source) of specimen - Strep A (or *S pyogenes*) identified: yes/no - Type of test - Care setting | - Other organisms involved yes/no.   - If yes, specify (e.g., *Staphylococcus aureus*, group B *Streptococcus*, *S. agalactiae*) - Anti-streptolysin O (ASO) and anti-DNAase B antibodies - Storage/transport identification number - Place/site of transfer of isolate for additional testing - Further testing ordered (e.g., *emm* typing, whole genome sequencing, anti-streptococcal antibody titers, speciation of large-colony β-hemolytic *Streptococcus*, antibiogram, etc.) |

Notes: ICU = Intensive Care Unit; LTCF=long-term care facility; *Mark unknown if cultures are conducted post administration of antibiotics.

## References

1. Okello E, Ndagire E, Muhamed B, et al. Incidence of acute rheumatic fever in northern and western Uganda: a prospective, population-based study. *The Lancet Global Health.* 2021;9(10):e1423-e1430.

2. Johnson DR, Kaplan EL, Bicova R, et al. *Laboratory diagnosis of group A streptococcal infections.* World Health Organization; 1996.

3. Spellerberg B, Brandt C. Laboratory diagnosis of Streptococcus pyogenes (group A streptococci). *Streptococcus pyogenes: Basic Biology to Clinical Manifestations [Internet].* 2016.

4. Kapatai G, Coelho J, Platt S, Chalker VJ. Whole genome sequencing of group A Streptococcus: development and evaluation of an automated pipeline for emmgene typing. *PeerJ.* 2017;5:e3226.

5. Solares JRA, Raimondi FED, Zhu Y, et al. Deep learning for electronic health records: A comparative review of multiple deep neural architectures. *Journal of Biomedical Informatics.* 2020;101:103337.

6. Wang Z, Shah AD, Tate AR, Denaxas S, Shawe-Taylor J, Hemingway H. Extracting diagnoses and investigation results from unstructured text in electronic health records by semi-supervised machine learning. *PLoS One.* 2012;7(1):e30412.

7. Arakaki RY, Strazzula L, Woo E, Kroshinsky D. The impact of dermatology consultation on diagnostic accuracy and antibiotic use among patients with suspected cellulitis seen at outpatient internal medicine offices: a randomized clinical trial. *JAMA Dermatology.* 2014;150(10):1056-1061.

8. World Health Organization. Surveillance standards for vaccine-preventable diseases. 2018.

9. World Health Organization. A definition for community-based surveillance and a way forward: results of the WHO global technical meeting, France, 26 to 28 June 2018. *Eurosurveillance.* 2019;24(2).

10. Deutscher M, Van Beneden C, Burton D, et al. Putting surveillance data into context: the role of health care utilization surveys in understanding population burden of pneumonia in developing countries. *Journal of Epidemiology and Global Health.* 2012;2(2):73-81.

11. U.S. Department of Health and Human Services. *E6(R2) Good Clinical Practice: Integrated Addendum to ICH E6(R1) Guidance for Industry* Maryland2018.
